# In Vitro Evaluation of the Effect of Different Luting Cements and Tooth Preparation Angle on the Microleakage of Zirconia Crowns

**DOI:** 10.1155/2021/8461579

**Published:** 2021-08-06

**Authors:** Behnaz Ebadian, Amirhossein Fathi, Melika Savoj

**Affiliations:** ^1^Dental Implants Research Center, Department of Prosthodontics, School of Dentistry, Isfahan University of Medical Sciences, Isfahan, Iran; ^2^Dental Material Research Center, Department of Prosthodontics, School of Dentistry, Isfahan University of Medical Sciences, Isfahan, Iran; ^3^Dental Students Research Committee, School of Dentistry, Isfahan University of Medical Sciences, Isfahan, Iran

## Abstract

**Introduction:**

Discrepancy between the crown border and prepared tooth margin leads to a microleakage that eases the penetration of microorganisms and causes the dissolution of luting cement consequently. Several factors should be considered to achieve optimal fitness, including tooth preparation taper and type of cementing agent. The study aimed to determine the relation of tooth preparation taper and cement type on the microleakage of zirconia crowns.

**Materials and Methods:**

Fifty-six freshly extracted premolars without caries and restorations were selected as the study sample and divided into two groups of different tapering degrees (6 and 12 degrees). Zirconia copings were designed and fabricated by the CAD/CAM system. The samples were divided into four subgroups for cementation, and each subgroup was cemented with a different luting cement (*n* = 7). After 5000 thermocycles at 5°C–55°C and dye penetration, the specimens were sectioned in the mid-buccolingual direction, and a digital photograph of each section was taken under a stereomicroscope. Data were analyzed by the Kruskal–Wallis and Mann–Whitney tests (*α* = 0.05).

**Results:**

The results showed significant differences among the four types of luting cement in marginal permeability (PV < 0.001). Regardless of the type of cement, the 12-degree tapering resulted in a lower microleakage (46.4% without microleakage) with statistically significant differences from the 6-degree tapering (PV = 0.042).

**Conclusion:**

Within the limitations of this study, increasing the tapering degree of the prepared tooth for CAD/CAM zirconia copings improved the marginal fit and decreased the microleakage score. In addition, total-etch resin cement indicated the least microleakage.

## 1. Introduction

The past decade was an outstanding era for developing dental materials, especially the rapidly increasing use of zirconia materials and metal-free dentistry, which provide high biocompatibility, enhanced esthetics, and improved material strength. The extensive knowledge gained about zirconia ceramic chemistry, crystallography, and engineered ceramics has led to advanced dental applications [1].

There is a controversy over the survival rate of ceramic crowns versus metal-ceramic crowns. Literature reviews concerning monolithic ceramic and metal-ceramic restorations have revealed that ceramic crowns with increased stability have shown survival rates similar to those of traditional porcelain-fused-to-metal (PFM) crowns [2]. In contrast, another study demonstrated that the survival of all-ceramic fixed dental prostheses (FDPs) was notably lower than that of PFM crowns [3].

Several factors mark the long-term success of the full-ceramic restorations, which a clinician should consider. The accurate marginal and internal fit of prosthetic crowns, along with high mechanical strength, good interfacial adhesion to the veneering material, and appropriate luting cement, are essential requirements for achieving the goal [4, 5]. Dentin hypersensitivity, dental caries, secondary caries, cement dissolution, plaque accumulation and retention, and periodontal inflammation are consequences of inadequate marginal fitness [6–8]. An idyllic marginal fit with no gap cannot yet be achieved because of the clinical and material-based errors [6]. Several authors agree that micro-infiltration is associated with the marginal discrepancy, where the most significant amount of cement is dissolved. It is essential to mention that the cementing agent is a critical factor in the longevity of restoration so that the characteristics and properties of the cement are drastically essential to prevent microleakage and attain a proper marginal fit [9–11].

The evaluation of microleakage results acquired with zinc phosphate, glass-ionomer, and resin cements has demonstrated zinc phosphate cement is less successful in decreasing microleakage than glass-ionomer and resin cements. A possible explanation may be on account of the fact that the zinc phosphate cement bond, which is exclusively mechanical, leads to higher solubility than glass-ionomer and resin cements [12, 13].

The resistance and retention forms of full crowns are ultimately affected by the convergence angle of the prepared teeth, which affects the adaptation of the fixed dental restoration. It has been suggested that a greater tapering degree allowing the increased thickness of all-ceramic crowns will increase their resistance to fracture [14]. Similarly, a larger axial convergence angle of the preparation should improve the marginal fit. [15].

The best index to determine the vertical and horizontal marginal misfit is an absolute marginal discrepancy (AMD). However, standardization of marginal discrepancy is not possible. AMD is the marginal gap measured between the axial wall of the tooth preparation and the margin of the crown in combination with extension error. There are two standard methods for measuring the microleakage, invasive (by sectioning the dye) and noninvasive (the direct view technique) techniques. In the invasive approach, which has been chosen for this study, dye penetration with different chromatic solutions such as safranin, methylene blue, and fuchsine is observed precisely under stereomicroscopes; consequently, this technique is considered to be more accurate than the noninvasive method [16].

A previous study by Emtair et al. [15] concerning the effect of axial convergence on the marginal fit of CAD/CAM zirconia copings showed that marginal fitness was improved by increasing the tapering degree of prefabricated dies. Marginal fit and microleakage of monolithic zirconia crowns cemented by bioactive and glass-ionomer cement were compared in a study by Aboelenen et al. [17]. They found that similarity in the physical properties and chemical composition of the two types of cement resulted in a nonsignificant effect on the extent of microleakage.

However, these studies have been done on prefabricated stainless-steel dies to compare preparation angles or other cement types. Despite the results obtained, controversial theories regarding the permeability and sealing function of cement and inadequacy of research in comparing both luting cement and preparation angles on natural teeth have made further evaluation necessary. Hence, this study aimed to determine the relation of the tapering degree and cement's type on the microleakage score of zirconia crowns on natural teeth. The null hypothesis to be tested is that there is no difference between microleakage observed in zirconia crowns with different preparation angles and types of cement.

## 2. Materials and Methods

This is an experimental in vitro study reviewed and approved by the university's ethics committee under study protocol (IR.SUMS.DENTAL.REC.1398.475).

### 2.1. Sample Collection and Preparation

Fifty-six maxillary and mandibular premolars extracted for orthodontic reasons were collected for this study. Written informed consent was obtained from the parents at the time of tooth extraction. The parents were informed about the purpose of the study, privacy preservation, and data anonymity. Teeth with any sign of caries and restorations were excluded from the study. The specimens were kept in a 5% sodium hypochlorite diluted solution for 10 minutes and rinsed with physiologic saline for debridement. Then, all teeth were stored in 3.3% Cetrimide-Chlorhexidine as a disinfectant solution at room temperature until preparation. For easy axial reduction procedure and preferable placement of the prepared specimen, the specimens were randomly selected and mounted in a former rubber cast base filled with type IV stone plaster (Vel-Mix; Kerr Corp, CA, USA) and extended 2 mm below the CEJ. The casts were divided equally into groups A and B (*n* = 28 per group) of different tapering degrees (6° and 12°) for reduction. Impressions were taken from the specimens using silicone putty (Speedex putty; Coltene, Altstatten, Switzerland) as an index for determining the amount of reduction. Occlusal reduction and flattening were performed by a round-ended tapered diamond bur (Tizkavan, Iran) up to 1.5–2 mm depth. One-mm cut depth grooves, axial reduction, and chamfer finishing line preparation were made by a 0.01 chamfer bur. Then, tapering modification was done by two different round-end tapered burs with two different tapering degrees (3° and 6°). To achieve the maximum accuracy for the occlusal convergence degree, the operator used a parallelometer (Dentsply Ceramco, Dentsply Sirona, USA) attached to a handpiece (Marathon, Escort-III, Daegu, South Korea). All sharp margins were rounded in the last step, and specimens were checked with putty index.

### 2.2. Coping Fabrication

All 56 specimen dies were digitalized by a laser scanner (3Shape A/S Copenhagen, Denmark), and zirconia copings (Ivoclar Vivadent, Schaan, Liechtenstein) were designed by the CAD system (350i imes-icore; Renfert GmbH, Eiterfeld, Germany) strictly following the manufacturer's protocol, whereby the 0.5 mm wall thickness and 30 *μ*m internal gap were applied. These results were transmitted to the laboratory to be executed by the CAM unit. Before cementation, final cleaning with pumice paste and water rinsing was done for all teeth to achieve a better bonding strength.

### 2.3. Crown Cementation

Each group (A and B) was divided into four subgroups. For each subgroup, a different luting cement was used (*n* = 7 per subgroup) (Table 1).

All cementations were performed by one operator at the same room temperature (25°C). Four types of cement were prepared as per the manufacturer's instructions:

  Group A and B subgroup I: self-adhesive resin cement (RelyX™ Unicem2 Self-Adhesive Universal Resin Cement Automix™; 3M ESPE, St. Paul, MN, USA) was applied after sandblasting the specimen with aluminum oxide. The cement was dispensed directly onto the bonding surface of the restoration.
  Group A and B subgroup II: dual-cure resin cement (RelyX™ Ultimate adhesive cement; 3M ESPE, St. Paul, MN, USA) was injected onto the crown and cured by the curing light for 20 seconds after overlaying, but before cementing, this group of teeth was etched with phosphoric acid (Ultra-Etch) for 15 seconds and rinsed for 10 seconds, following which the bond (Scotchbond™ Universal Adhesive; 3M ESPE, St. Paul, MN, USA) was applied for 20 seconds.
  Group A and B subgroup III: glass-ionomer cement (GC Gold Label self-cured luting and cement; GC, Tokyo, Japan) was obtained by mixing one spoonful of powder and two drops of liquid. The company's recommendation is to divide the powder into two parts. Two drops of liquid were mixed with one part for 10 seconds on a vast area of the mixing pad before adding the second part. The paste was inserted into the crown with a spatula (mix and place spatula #24M; Nordent®, USA), and the crown was placed on the abutment.
  Group A and B subgroup IV: zinc phosphate cement normal setting (Hoffmann Dental Manufacturer GmbH, Berlin, Germany) was prepared with 1.5 g powder and 1.0 g liquid for fixation consistency. The powder was divided into four portions (1/2-1/4-1/8-1/8). Mixing was started with the minor portion and ended in 90 seconds. Then, the paste was placed into the crown with a spatula, and the crown was placed on the abutment.

After crown positioning, a vertical force of 5 kg was applied to it by an apparatus loading for 10 minutes. The excessive amount of cement was removed by a sharp curette. As the setting time of all groups finished, all teeth were placed in distilled water at 37°C for 24 hours. They were then thermocycled (Delta Tpo2, Iran) at 5°C–55°C for 5000 cycles with a dwell time of 20 seconds to stimulate the oral condition for one year. Before dye penetration, the specimens were dried, and the root surfaces were covered with two layers of acrylic fingernail polish 1 mm below the crown margin to prevent dye penetration into other areas of the specimens.

### 2.4. Dye Penetration and Microleakage Evaluation

The samples were immersed in 0.5% basic fuchsine solution and placed in a shaking incubator (Pars Azma, Iran) for 48 hours at 37°C to shake them every 4 hours and prevent the sedimentation of the solution. After rinsing with distilled water and drying, the coronal parts of the specimens were embedded in clear cold cure acrylic resin (PROCAST DSP clear shade; GC, Tokyo, Japan). Then, all roots were cut off using a diamond disk for easy handling. The embedded crowns were sectioned in the mid-buccolingual or mid-buccopalatal direction using a water-cooling saw (Nonstop, Iran). A digital photograph of each section was taken under a stereomicroscope (Trinocular Zoom Stereo Microscope, SMP200; HP, USA) at an original magnification of 20×. The extent of dye penetration into the surface of the section was evaluated and recorded by one operator according to the following scores [18]:

  0: no evidence of dye penetration at the tooth-restoration interface
  1: dye penetration up to one-third of chamfer preparation
  2: dye penetration up to two-thirds of chamfer preparation
  3: dye penetration along all of the chamfer preparation
  4: dye penetration greater than one-third of the axial wall
  5: dye penetration greater than two-thirds of the axial wall
  6: dye penetration along all of the axial walls, including the occlusal edge

### 2.5. Statistical Analysis

The statistical analysis was performed using SPSS version 23.0 (SPSS Inc., Chicago, IL, USA). Cement's comparison was analyzed using the Kruskal–Wallis test, and preparation angle and combination of cement-preparation angle results were evaluated by the Mann–Whitney test. The level of significance was considered as *P* < 0.05.

## 3. Results

Microleakage scores are demonstrated in Table 2. As a result of the Mann–Whitney test, regardless of the type of cement, the 12-degree tapering resulted in a lower microleakage with statistically significant differences from the 6-degree tapering (PV = 0.042). Based on the resulted *P* values of the Kruskal–Wallis test (PV = 0.007) (PV = 0.006), there is a significant difference at least between two cement types in marginal permeability with either preparation degrees (Table 3). The pairwise comparisons between types of cement using the Mann–Whitney test showed a significant difference in two out of the six groups (Table 4). Hoffmann's cement presented the highest microleakage scores compared with Ultimate and Unicem cements (*P* < 0.05). GC-Ultimate also showed a difference (*P*=0.055), but it was not statistically significant.

**Figure 1:**
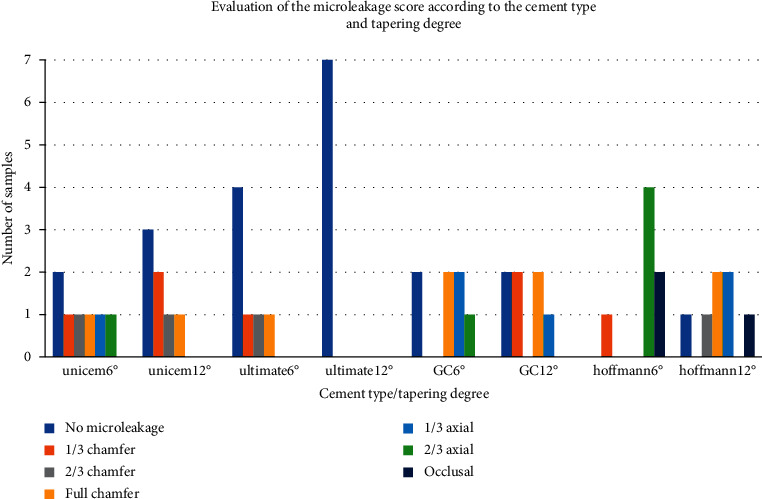
Evaluation of the microleakage score according to the cement type and tapering degree.

Figure 1 illustrates the two different tapering degrees and four types of cement to show the frequency distribution of the microleakage score.

## 4. Discussion

The present study measures the relation of the tapering degree on the microleakage score of zirconia crowns on natural teeth, as well as the permeability of self-adhesive resin, dual-cure resin, glass-ionomer, and zinc phosphate cements. The study hypotheses were partially rejected because the type of cement and preparation angel influences the die penetration and microleakage score to some extent.

During the past two decades, the popularity and utilization of zirconium restorations have increased despite economic concerns owing to their esthetics, color stability, and higher strength than other types of ceramics. The manufacturing accuracy of these restorations is also crucial in preventing microleakage [19–22]. However, it is still unclear whether or not the zirconia crowns are a valid alternative to classic metal-based crowns [23]. One of the challenges clinicians have faced over time is the discrepancy between crown border and prepared tooth margin, which leads to microleakage, eases the penetration of microorganisms, and causes the dissolution of luting cement [24]. Therefore, zirconia crown was implemented in this study to investigate their marginal adaptation along with other factors.

Numerous studies have stated a parallel relationship between the marginal gap width and post-treatment complications [14, 25, 26]. The convergence angle of the prepared teeth is another essential factor that affects the resistance and retention forms of crown restoration, and lack of each of these two is potentially detrimental to crown fitness, which can lead to microleakage. The results of a study on the influence of convergence angle and cement space on the adaptation of zirconia copings suggested that increasing the convergence angle of the abutment reduced the internal space [27]. Beuer et al. [28] aimed to evaluate the effect of preparation angels on the accuracy of zirconia copings. They concluded that the 12-degree occlusal convergence could eventually lead to the best precision in single zirconia crowns compared with tapering degrees of 4 and 8. This result tied well with the current study, where the tapering degree of 12 achieved the best overall score in microleakage. Regardless of the cement type, these findings are in agreement with conducted research. In this study, the tapering degree of 12 resulted in the least microleakage with statistically significant differences from the tapering degree of 6 (*P* value = 0.042). Nonetheless, there is a remarkable limitation in tooth preparation and reduction for esthetic crowns compared with preformed metal crowns.

Hence, to achieve superior adhesion and retention despite preparation limitations, the type and amount of luting cement play a role and should be deliberated to prevent microleakage [29, 30]. Effective cementation is critical to achieving the long-term success of crown sustainability, and poor seating leads to inadequate marginal adaptation [31]. Glass-ionomer resin cement and dual-cure resin cement could be considered as ideal cementing agents due to easy handling, reasonable cost, indissolubility in oral fluid, bonding strength, fluoride releasing, and the low score of microleakage [29, 32, 33]. However, the most commonly used cement is zinc phosphate, which has some disadvantages such as solubility and lack of bonding [34]. Regarding the longevity of zinc phosphate cement in the oral cavity, the present study confirmed the findings that zirconia crowns cemented with (Hoffmann) zinc phosphate cement shows a higher degree of microleakage than other types of cement. This could be validated based on the research by Al-Shakir et al. [29] in which the researchers attempted to evaluate the marginal adaptation of metal-ceramic crowns to different types of luting cement. They believed that the type of cementing agent affected the microleakage score. In addition, the results of the present study indicated no difference between glass-ionomer and zinc phosphate cements. This finding was also in accordance with previous research, which showed no significant difference for the mean cement thickness or microleakage score comparing glass-ionomer cement (GIC) and phosphate monomer containing resin cement (MDP-RC) under two types of zirconia crowns (manufactured by different procedures) [35].

Controversial studies regarding the effectiveness of self-etching (SE) in comparison with self-adhesive (SA) resin cement leads to an evaluation of a wide variety of these agents. Although a study by Cristian et al. [36] reported no tangible difference among various resin luting cement to prevent microleakage, an in vitro survey stated a significant difference in microleakage of (SE) in comparison with (SA) resin cement [20]. Contrary to the findings of an in vitro research regarding the correlation between microleakage and absolute marginal discrepancy in zirconia crowns cemented with four types of resin cement, the total/self-etch adhesive cement (RelyX Ultimate) showed the smallest value of microleakage. Nonetheless, the difference between the total-etch and self-adhesive cement was not statistically significant, in line with the mentioned study [36]. Al-Haj-Ali et al. [37] obtained a similar pattern in results despite the fact that the evaluation was performed on the metal crowns. The results showed that impermeability was higher in resin cement than in glass-ionomer cement. Even though the current research did not replicate the previously reported findings of the effect of types of cement on microleakage, our results suggest that self-etching resin cement has a higher ability in marginal sealing than self-adhesive resin cement. The present research results are in line with those of Owittayakul et al. [20] regarding the microleakage of phosphate monomer-based resin cement. It is worth mentioning that the present study was conducted on the extracted teeth, while the study of Owittayakul was carried on acrylic molars.

A difference between the findings of the present study and those of other previous ones can only be attributed to the sample size and type of teeth evaluated. However, the existing differences are broadly acceptable. Notwithstanding the limitations of this study, future studies could fruitfully explore this issue further by adding chewing stimulation along with thermocycling and evaluate in more clinically relevant settings. In addition, further work with bacterial penetration is undoubtedly required to confirm this finding, while the size of microorganisms is dissimilar to chromatic particles.

## 5. Conclusion

The null hypothesis stating that there is no difference between microleakage observed in zirconia crowns with different preparation angles and types of cement is partially rejected. There was a significant difference among the four types of luting cement in marginal permeability, and RelyX Ultimate showed the least microleakage. Regardless of the type of cement, the 12-degree tapering resulted in a lower microleakage with statistically significant differences from the 6-degree tapering.

## Figures and Tables

**Table 1:** Classification and batch number of the tested cements.

Product	Type	Delivery system	Lot no.	Manufacturer
RelyX™ Unicem	Dual-cure self-adhesive resin cement	Automix syringe	5220265	3M ESPE, St. Paul, MN, USA
RelyX™ Ultimate	Dual-cure self-etch or total-etch resin cement	Automix syringe	5238608	3M ESPE, St. Paul, MN, USA
GC Gold Label	Self-cure glass-ionomer cement	Powder/liquid	180425D	GC Dental, Tokyo, Japan
Hoffmann's cement	Self-cure zinc phosphate cement	Powder/liquid	7670	Hoffmann Dental Manufacturer GmbH, Berlin, Germany

**Table 2:** Microleakage scores.

	Number	Mean	Median	SD	Range
Unicem-6°	7	2.14	2.00	1.952	5
Unicem-12°	7	1.00	1.00	1.155	3
Ultimate-6°	7	0.86	0.00	1.215	3
Ultimate-12°	7	0.00	0.00	0.000	0
GC-6°	7	2.71	3.00	1.976	5
GC-12°	7	1.71	1.00	1.604	4
Hoffmann-6°	7	5.00	5.00	2.000	6
Hoffmann-12°	7	3.29	3.00	2.138	7
Total	56	2.09	1.50	2.143	7

**Table 3:** Frequency of microleakage scores in each experimental condition (*n* = 7).

Tapering degree	Microleakage score	Unicem, *n* (%)	Ultimate, *n* (%)	GC, *n* (%)	Hoffmann, *n* (%)	*P* value (Kruskal–Wallis)
6**°**	0	2 (28.6)	4 (57.1)	2 (28.6)	0	.007
	1	1 (14.3)	1 (14.3)	0	1 (14.3)	
	2	1 (14.3)	1 (14.3)	0	0	
	3	1 (14.3)	1 (14.3)	2 (28.6)	0	
	4	1 (14.3)	0	2 (28.6)	0	
	5	1 (14.3)	0	1 (14.3)	4 (57.1)	
	6	0	0	0	2 (28.6)	

12**°**	0	3 (42.9)	7 (100)	2 (28.6)	1 (14.3)	.006
	1	2 (28.6)	0	2 (28.6)	0	
	2	1 (14.3)	0	0	1 (14.3)	
	3	1 (14.3)	0	2 (28.6)	2 (28.6)	
	4	0	0	a1 (14.3)	2 (28.6)	
	5	0	0	0	0	
	6	0	0	0	1 (14.3)	

*P* value (Mann–Whitney)		0.318	0.314	0.447	0.190	

**Table 4:** Results of the Mann–Whitney *U* test for each subgroup (*P* value).

	6 degrees	12 degrees
Unicem-Ultimate	0.217	0.214
Unicem-GC	0.660	0.449
Unicem-Hoffmann	0.031^*∗*^	0.002^*∗*^
Ultimate-GC	0.094	0.055
Ultimate-Hoffmann	0.001^*∗*^	0.002^*∗*^
GC-Hoffmann	0.086	0.243

^*∗*^A significant difference in microleakage score (*P* value  < 0.05).

## Data Availability

The data used to support the findings of this study are available from the corresponding author upon request.
